# Associations Between Aerobic Fitness and Cognitive Control in Adolescents

**DOI:** 10.3389/fpsyg.2018.01298

**Published:** 2018-08-14

**Authors:** Daniel R. Westfall, Anne K. Gejl, Jakob Tarp, Niels Wedderkopp, Arthur F. Kramer, Charles H. Hillman, Anna Bugge

**Affiliations:** ^1^Department of Psychology, Northeastern University, Boston, MA, United States; ^2^Centre of Research in Childhood Health, Institute for Sport Science and Clinical Biomechanics, University of Southern Denmark, Odense, Denmark; ^3^Sports Medicine Clinic Orthopedic Department, Institute of Regional Health Research, Middelfart Hospital, University of Southern Denmark, Middlefart, Denmark; ^4^Beckman Institute, University of Illinois, Urbana, IL, United States; ^5^Department of Health Sciences, Northeastern University, Boston, MA, United States

**Keywords:** fitness, inhibitory control, cognitive flexibility, diffusion modeling, executive function

## Abstract

Previous research has found positive associations between cognitive control and aerobic fitness in preadolescents and adults; however, fewer studies have investigated these associations in adolescents. Adolescence is of particular interest due to continued maturation of the prefrontal cortex; an area that subserves cognitive control. This study investigated the associations of aerobic fitness and cognitive control in adolescents. An assessment of aerobic fitness (Andersen intermittent running test) and two tests of cognitive control were collected to investigate these associations. Participants completed a test of inhibitory control (flanker task) and a test of cognitive flexibility (switch task). Along with traditional measures of reaction time (RT) and accuracy, diffusion modeling was utilized to combine these measures to calculate latent variables (i.e., drift rate, boundary separation, and nondecision time). Associations between cognitive measures and fitness were assessed with linear regressions while controlling for potential confounding factors. Higher fitness was associated with shorter reaction time and higher accuracy in the flanker task, indicating better inhibitory control performance. In addition, greater aerobic fitness was associated with greater quality of information uptake in the flanker task, as indicated by drift rate. In the switch task, higher aerobic fitness was associated with greater accuracy and longer switch RT indicating a speed-accuracy tradeoff. Results from the switch task diffusion modeling supported this conclusion as indicated by greater fitness associated with greater boundary separation, or response conservativeness. Further, greater drift rate in the switch task was associated with greater fitness. These findings corroborate growing evidence indicating the importance of aerobic fitness for inhibitory control and cognitive flexibility. This study extends the literature by demonstrating these effects in a large sample of adolescents with a computational model of the mechanisms that underlie cognition.

## Introduction

An emerging body of literature has demonstrated the importance of aerobic fitness for cognition and brain health across the lifespan (Themanson et al., 2008; Chaddock-Heyman et al., 2014; Erickson et al., 2015; Hwang et al., 2017). Therefore, reports on decreased levels of physical activity and increased participation in sedentary behaviors among children and adolescents (Ekelund et al., 2011) coinciding with trends of decreasing aerobic fitness (Tomkinson et al., 2003) are alarming. Indeed, research indicates that these trends might affect not only metabolic health (Katzmarzyk et al., 2009; Ekelund et al., 2012), but also cognition and brain health (Vaynman and Gomez-Pinilla, 2006). Much of the current literature on the aerobic fitness, cognition, and brain health relationship has focused on the extremes of the lifespan (i.e., preadolescent children and older adult populations), while fewer studies have focused on adolescence (for exceptions see Stroth et al., 2009; Herting and Nagel, 2012; Hogan et al., 2013; Huang et al., 2015). However, adolescence might be of particular interest because of the possibility of influencing the course of brain development during a period of the lifespan characterized by rapid development and maturation.

Importantly, brain maturation is not uniform, as specific regions, such as prefrontal cortex and hippocampus, are found to exhibit protracted maturation (Luna, 2009). Consequently, cognitive functions subserved by these regions and their associated networks also exhibit protracted development. Specifically, cognitive control (also known as executive function or executive control) is mediated by the prefrontal cortex and reaches maturity during the third decade of life (Luna, 2009). Cognitive control has been associated with educational outcomes (St Clair-Thompson and Gathercole, 2006), health behaviors (Hillman et al., 2015), and health outcomes (Moffitt et al., 2011). It refers to top-down, goal-directed behavior and is composed of inhibitory control (the ability to ignore distracting information and focus on task relevant information), working memory (the ability to hold and manipulate information for a brief period of time), and cognitive flexibility (the ability to shift attention or alter response strategies in response to changing task demands) (Miyake et al., 2000; Lehto et al., 2003). While preadolescents are capable of completing tasks that measure cognitive control abilities; fine tuning of cognitive control continues throughout adolescence until a mature level of performance is achieved in early adulthood. Such fine tuning of cognitive control is thought to result from structural and functional changes that continue throughout this time; including synaptic pruning, myelination, and integration of cortical areas (Luna, 2009). As such, adolescence is an important time period for the study of different aspects of cognitive control.

Among the reported associations of aerobic fitness and cognition, cognitive control has been found to be particularly related to aerobic fitness levels in both preadolescent children (Buck et al., 2008; Pontifex et al., 2011; Voss et al., 2011; Chaddock et al., 2012; Hillman et al., 2014, 2015) and adult populations (Colcombe and Kramer, 2003; Prakash et al., 2011; Verstynen et al., 2012; Erickson et al., 2015; Kawagoe et al., 2017), but only a few studies have focused on cognitive control in adolescence (Stroth et al., 2009; Hogan et al., 2013; Huang et al., 2015). Specifically, Hogan et al. (2013) reported that unfit participants had higher error rates compared to fit participants in a combined modified Eriksen flanker and Go/NoGo task during a rest condition. A larger cross-sectional study (*N* = 525) by Huang et al. (2015) found that higher aerobic fitness was associated with shorter reaction time (RT) with no association for response accuracy in a modified Eriksen flanker task. However, Stroth et al. (2009) did not observe behavioral differences between unfit and fit participants in a combined modified Eriksen flanker and Go/NoGo task. As such, the current adolescent literature investigating aerobic fitness and cognitive control associations lacks consensus.

To date, most studies have used typical task performance measures (i.e., RT and accuracy) to assess the relationship between aerobic fitness and cognition. Apart from these measures, a signal detection theory method known as diffusion modeling (Ratcliff, 1978) will provide additional information to elucidate the associations between aerobic fitness and cognitive control. Whereas traditional analyses investigate RT and accuracy separately, this computational model allows for the integration of these outcomes to investigate latent variables that contribute to these behavioral measures. In other words, accuracy and RT information is combined and distilled into separable underlying processes, which contribute to the accuracy or RT outcome measures. Specifically, drift rate refers to the rate of information uptake (i.e., the speed and quality of stimulus information processing, a higher drift rate would indicate better performance). Boundary separation is a measure of response conservativeness and speed-accuracy tradeoff and therefore a measure of response strategy (i.e., a larger boundary separation results in greater accuracy but longer RTs). Finally, nondecision time refers to time spent in nondecision related processing (i.e., time spent during encoding, memory access, and response execution). Indeed, a 2015 review of the existing physical activity and cognition literature has called for the exploration of computational models that account for both RT and accuracy effects, such as diffusion modeling (Prakash et al., 2015). Diffusion modeling has been important for exploring nuanced differences in cognitive control between older adults and children. Both age groups exhibit longer RTs compared to young adults, but children have reduced uptake of quality information (i.e., drift rate; Ratcliff et al., 2012), whereas older adults exhibit more response conservativeness (i.e., boundary separation; Ratcliff et al., 2004). To our knowledge, no studies have applied diffusion modeling to aerobic fitness and cognitive control in adolescents. Results from these measures might help elucidate the specific latent variables (i.e., drift rate, boundary separation, and nondecision time) of cognitive control that are associated with aerobic fitness in adolescents.

Accordingly, the purpose of the present study was to explore associations of aerobic fitness and cognitive control in a large adolescent population. A modified flanker task (Eriksen and Eriksen, 1974; Hillman et al., 2009b) was used to measure inhibitory control and a switch task was used as a measure of cognitive flexibility (Espy, 1997; Cepeda et al., 2001; Hillman et al., 2014). Both traditional analyses (i.e., analysis of accuracy and RT) and diffusion modeling were performed across tasks. Based on the literature with preadolescent children (e.g., Hillman et al., 2014), we hypothesized that higher levels of aerobic fitness would be associated with better task performance (i.e., greater accuracy and shorter RTs), particularly during task conditions that require greater amounts of cognitive control (i.e., inhibitory control and cognitive flexibility). Associations between aerobic fitness and specific measures obtained through diffusion modeling remain unexplored in adolescents. However, because children demonstrate reduced uptake of quality information (i.e., reduced drift rate), we further hypothesized that drift rate would be particularly sensitive to aerobic fitness in the studied adolescence age group (Ratcliff et al., 2012).

## Methods

### Population and study design

This manuscript is based on data from the third major data collection in the Childhood Health, Activity, and Motor Performance School Study Denmark (the CHAMPS study-DK 3) and the analyses are therefore cross-sectional. The CHAMPS study-DK originally focused on motor skill development, metabolic health, and musculoskeletal injuries. The study was initiated in 2008, when all 19 schools in the municipality of Svendborg were invited to participate in the study. The full study description has been described in detail previously (Wedderkopp et al., 2012). Therefore, only methods pertinent to this paper will be described. In 2014, all children and parents/legal guardians received information about the planned follow-up in 2015 through school meetings and written information. Parents/legal guardians of 745 children or adolescents provided written informed consent to participate in the CHAMPS study-DK 3. Only participants with an acceptable aerobic fitness test and at least one acceptable cognitive test (see section Cognitive Tasks for exclusion criteria) in 2015 were included in the current study yielding a total sample-size of 610 participants (Table 1). The overwhelming majority of missing cognitive tests owed to school non-attendance. As such, an initial 568 participants completed the flanker task and the measure of aerobic fitness, of these 14 were removed as outliers (see Cognitive Tasks section for criteria), resulting in a sub-sample of 554 participants. An initial 565 participants completed the switch task and had aerobic fitness information, of these 42 were removed as outliers (see Cognitive tasks section for criteria) resulting in a sub-sample of 523 participants. The Regional Committees on Health Research Ethics for Southern Denmark (Project number: S-20080047 and S-20140105) approved the study.

**Table 1:** Demographic Information, mean, and SD by sex.

	**Males**		**Females**	
**Measure**	**Value**	**SD**	**Value**	**SD**
N	310	–	300	–
Age (years)^*^	14.35	1.25	14.06	1.27
Aerobic Fitness (meters)^*^	1139.21	106.05	1041.83	93.65
PUBERTAL TIMING, N (%)^*^				
Stage 1 and 2	36 (11.7)	–	14 (4.6)	–
Stage 3	86 (27.7)	–	124 (41.6)	–
Stage 4	130 (41.9)	–	135 (45.3)	–
Stage 5	58 (18.7)	–	25 (8.4)	–
MOTHER'S EDUCATION, N (%)^**^				
Unknown	13 (4.2)	–	18 (6.0)	–
10th grade or less	3 (1.0)	–	9 (3)	–
High school	20 (6.5)	–	16 (5.3)	–
Vocational	93 (30.0)	–	80 (26.7)	–
Short tertiary	37 (11.9)	–	32 (10.7)	–
Bachelor or equiv	121 (39)	–	124 (41.3)	–
Masters or equiv	23 (7.4)	–	21 (7.0)	–

* *Significantly different between males and females, p < 0.05*.

** *Mother's education was provided by mother or female guardian*.

### Anthropometrics

Weight was measured to the nearest 0.1 kg on an electronic scale (Tanita BWB-800S, Tanita Corporation, Tokyo, Japan) with children in light clothing. Height was measured to the nearest 0.5 cm using a portable stadiometer (Harpenden stadiometer, West Sussex, UK). BMI was calculated as weight/height^2^ (kg/m^2^). Pubertal timing was self-assessed using the Tanner pubertal stages by examining pictures of pubic hair development for males and breast development for females (Tanner, 1981; Taylor et al., 2001), categories 1 and 2 were collapsed because of low reporting of category 1 (4 participants). Pubertal timing was missing for two participants with flanker data and one participant with switch data. To preserve these participants, a mean replacement was utilized. The same pattern of results occurred regardless of whether these participants were removed or included.

### Maternal education

The mother's or female guardian's highest completed education obtained from a questionnaire and categorized as follows; completion of: (1) 10th grade or less, (2) vocational education, (3) high school education, (4) short tertiary education, (5) bachelor's degree or equivalent, (6) master's degree or higher. If the 2015 questionnaire was not available, information about parental education from the most recent questionnaire from 2012 or 2008 were used. If mother's education was missing then information on father's education was used (3 participants). If either of the parent's education information was unavailable then this variable was coded as a missing category (23 participants with flanker data; 21 participants with switch data). The primary analysis included the missing category. A re-analysis removing participants with missing maternal education did not alter the results for the variables of interest.

### Aerobic fitness

Aerobic fitness was assessed by the Andersen test, an intermittent running test that has been validated against direct measures of maximum oxygen uptake in different age groups and found to be both valid and reliable (Andersen et al., 2008). Criterion validity against maximal oxygen uptake (*r*^2^ approximately 0.5) and test-retest reliability (*r*^2^ approximately 0.7–0.8) are available from independent, albeit younger, samples (Ahler et al., 2012; Aadland et al., 2014). In this test, participants are instructed to run between two lines 20 meters apart and touch the floor behind each line before turning around and continuing to run. Running continues for 15 s, then they are instructed to stop immediately and stand for 15 s, afterwards they continue running between the two lines. This pattern continues for 10 min. Total distance was used as the measure of aerobic fitness. The test leader could subjectively judge the test “not accepted” if a participant stopped before the test was ended or appeared to deliberately perform poorly (e.g., talking or showing no sign of physical exertion).

### Cognitive tasks

To assess cognitive control in the areas of inhibitory control and cognitive flexibility, two different computer-based tasks were used. Specifically, a modified Eriksen flanker task (Eriksen and Eriksen, 1974) and a color-shape switch task (Espy, 1997; Hillman et al., 2014) were performed to assess inhibition and flexibility, respectively. In both tasks participants were instructed to respond as quickly and accurately as possible. Measures of RT and response accuracy were collected. The modified flanker task consists of trials of five arrows on a screen. Participants were instructed to respond to the directionality of a central target stimulus. A target arrow pointing to the right “>” required a right-handed response and a target arrow pointing to the left “<” required a left-handed response. Responses were made with an index finger press on a keyboard. Flanking arrows could be either congruent (>>>>> or <<<<<), or incongruent (>><>> or <<><<) to the target arrow. Congruent trials require lower amounts of inhibitory control to select the correct response as only one response mapping is activated. However, incongruent trials require higher levels of inhibitory control resulting in longer RTs and lower accuracy due to the activation of both response mappings, of which one must be inhibited in favor of the correct response mapping. Before the task, participants completed a practice block of 20 trials. The test consisted of two blocks of 75 presentations with congruent and incongruent trials being presented randomly and with equal probability. Stimuli were presented for 120 ms with a response window of 1,350 ms and a variable waiting period of 1,250, 1,350, 1,450, or 1,550 ms between trials. A break of 30 s separated each block. Response accuracy was defined as the percentage of correct responses, while RT was defined as the average time to response selection on correct trials. Further, differences in accuracy and RT between congruent and incongruent conditions are presented as interference-scores (i.e., accuracy interference = congruent–incongruent; RT interference = incongruent–congruent). Participants who had an overall accuracy of < 50% (8 participants) or were coded as an RT outlier (>3 SD above or below the mean in the outcome variables; 6 participants) were discarded from further analysis.

The color-shape switch task consisted of characters of different shapes (square or circle) and colors (blue or green) presented on a screen. In the first part, participants completed the homogeneous condition and were instructed to respond to either shape or color in separate blocks (e.g., a left response to square and a right response to circle; or a left response to green and a right response to blue). After completing the homogeneous condition, participants completed the heterogeneous task condition and were instructed to flexibly switch their responses between shape and color based upon a cue (whether the character had its arms up or down; e.g., when the arms are up they complete the shapes task and when the arms are down they complete the color task). The heterogeneous condition required them to hold two rule sets in working memory and activate the correct rule set based on the cue. Each block was separated by a 30 s break and was preceded by 20 practice trials. The homogenous blocks (single rule set) consisted of 50 trials each and the heterogeneous block (consisting of both rule sets) had 104 trials with equiprobable switch and non-switch trials. The heterogeneous block consisted of two trial types: non-switch trials, where the previous trial (*n*−1) and the current trial (*n*) did not change response sets; or switch trials, where the previous trial (*n*−1) and the current trial (*n*) changed response sets. Stimuli were presented for 250 ms with a response window of 1,950 ms and a waiting period of 2,000 ms between trials. Response accuracy was defined as the percentage of correct responses while average RT was defined as time to respond on correct trials. Participants who had an overall accuracy of < 50% (38 participants) or were coded as an RT outlier (>3 SD above or below the mean in the outcome variables; 4 participants) were discarded from further analysis. Additional performance measures were calculated as local switch cost (the cost of switching between response sets, presented as the difference in performance between non-switch and switch trials) and global switch cost (the difference in performance due to the difficulty of maintaining multiple rule sets in working memory, presented as the difference in performance between homogeneous and heterogeneous blocks). Local switch cost was calculated as switch–non-switch for RT and non-switch–switch for accuracy. Global switch cost was calculated as heterogeneous–homogeneous blocks for RT and homogeneous–heterogeneous blocks for accuracy.

### Diffusion modeling

Accuracy, correct response RT, and correct response RT variance data were subjected to the EZ-Diffusion model (see Wagenmakers et al., 2007 for methods), which allows for the calculation of drift rate, boundary separation, and nondecision time. Drift rate refers to the amount of information uptake per time unit or to the slope of the diffusion process. Boundary separation is a measure of response conservativeness and speed-accuracy tradeoff. Nondecision time is the amount of time spent on all nondecision processing and incorporates the preparatory and encoding processes preceding the decisional phase and motor activation of the response (Ratcliff, 1978; Wagenmakers et al., 2007). The advantage of the EZ-Diffusion model is that it allows for estimation of these parameters without requiring large amounts of error responses or experimental trials (Wagenmakers et al., 2007).

### Statistical analyses

Descriptive characteristics were summarized by sex. Analyses were run to determine any differences in demographics between sexes and between subjects included and excluded in the two main analyses (flanker and switch task). For continuous variables, the differences were evaluated using unpaired *t*-tests and for categorical variables using Chi-square tests, and *post-hoc* standardized residuals were calculated to determine which individual cells contributed to the omnibus chi-square value (Beasley and Schumacker, 1995).

Initial Spearman's rank order correlation analyses (Table 2) were conducted to identify covariates for the inclusion in the regression analysis, covariates were identified at *p* < 0.05. Dependent measures (accuracy and RT) from the flanker (congruent, incongruent, interference scores) and switch (homogeneous, non-switch, switch, global switch cost, local switch cost) tasks as well as the related diffusion measures for each (drift, boundary separation, nondecision time) were correlated with measures of fitness, age, pubertal timing, and mother's education. Hierarchical regression analyses were used to investigate the amount of variance in the dependent measures explained by aerobic fitness (Step 2), independent of the variance explained by the descriptive factors (Step 1). Because of important relationships between age and sex with aerobic fitness (Armstrong and Welsman, 2007), these variables were included in Step 1 with any additional covariates. Assumptions of independence, equality of variance, linearity, and normality were plotted, inspected, and verified using Studentized residuals. Multicollinearity was not observed among any of the regression analyses.

**Table 2:** Spearman Correlations Between Dependent and Demographic Variables and Mean values.

**Measure**	**Aerobic fitness**	**Age**	**Mother's education**	**Pubertal timing**	**Mean (SE)**
**FLANKER**					
Congruent RT	−0.113^**^	−0.222^**^	0.043	−0.169^**^	450 (2.3)
Incongruent RT	−0.126^**^	−0.211^**^	0.051	−0.157^**^	543 (3.2)
Congruent acc	0.099^*^	0.192^**^	0.081	0.032	95.6 (0.3)
Incongruent acc	0.079	0.208^**^	0.059	−0.011	81.7 (0.6)
Interference RT	−0.077	−0.084^*^	0.035	−0.058	93.4 (1.8)
Interference acc	−0.039	−0.146^**^	−0.025	0.034	13.9 (0.4)
Congruent drift	0.138^**^	0.297^**^	0.048	0.078	0.373 (0.005)
Incongruent drift	0.112^**^	0.296^**^	0.059	0.048	0.202 (0.004)
Congruent boundary separation	0.000	0.065	0.033	0.034	0.110 (0.001)
Incongruent boundary separation	−0.074	−0.144^**^	0.049	−0.125^**^	0.089 (0.001)
Congruent nondecision time	−0.029	−0.076	0.055	−0.166^**^	0.302 (0.002
Incongruent nondecision time	0.003	0.059	0.024	−0.010	0.379 (0.003)
**SWITCH**					
Homogeneous RT	−0.147^**^	−0.169^**^	0.044	−0.137^**^	458 (3.1)
Homogeneous acc	0.022	0.178^**^	0.060	−0.001	90.7 (0.3)
Non-switch RT	0.044	−0.088^*^	0.064	−0.148^**^	949 (11.7)
Switch RT	0.066	−0.052	0.070	−0.116^**^	1,146 (16.0)
Non-switch acc	0.193^**^	0.186^**^	0.053	0.041	81.0 (0.6)
Switch acc	0.214^**^	0.216^**^	0.080	0.035	75.9 (0.6)
Global switch cost RT	0.100^*^	−0.031	0.063	−0.111^*^	587 (12.5)
Local switch cost RT	0.072	0.027	0.049	−0.016	196 (7.6)
Global switch cost acc	−0.221^**^	−0.122^**^	−0.040	−0.045	12.4 (0.5)
Local switch cost acc	−0.048	−0.063	−0.048	0.007	5.1 (0.4)
Non-switch drift	0.179^**^	0.221^**^	0.042	0.096^*^	0.099 (0.002)
Switch drift	0.208^**^	0.254^**^	0.043	0.071	0.078 (0.002)
Non-switch boundary separation	0.120^**^	0.055	0.073	−0.057	0.169 (0.002)
Switch boundary separation	0.066	0.048	0.042	−0.047	0.165 (0.002)
Non-switch nondecision time	0.018	−0.096^*^	0.033	−0.112^**^	0.372 (0.006)
Switch nondecision time	0.113^**^	−0.009	0.065	−0.098^*^	0.556 (0.010)

* *p < 0.05*.

** *p < 0.01*.

## Results

### Demographic information

Table 1 summarizes the demographic information for all participants with available demographic information (*n* = 610).

#### Drop-out analyses

Investigations of the flanker subgroup included in the analyses (*n* = 554) with those excluded from analyses (*n* = 150) found that excluded individuals were slightly older compared to the subgroup included in the analyses (mean = 14.48, SD = 1.56 vs. mean = 14.15, SD = 1.17), *t*_(702)_ = 2.89, *p* = 0.015. There were significantly more girls than boys excluded from analyses compared to the group with flanker results (60% girls compared to 40% boys were excluded), *X*^2^ (1, *N* = 704) = 5.08, *p* = 0.024, and significantly different proportion of mother's education, *X*^2^ (7, *N* = 704) = 17.8, *p* = 0.013. Post-hoc analyses of the mother's education indicated that the excluded group had more participants with an unknown mother's education than predicted by the model compared to the included group.

The switch subgroup included in the analyses (*n* = 523) with those excluded from analyses (*n* = 181) found that excluded individuals were slightly younger [*X*^2^ (1, *N* = 704) = 4.04, *p* = 0.036], had a lower percentage of girls (58.6 vs. 49.5%), and a significantly different education distribution [*X*^2^ (7, *N* = 704) = 15.82, *p* = 0.027]. *Post-hoc* analyses indicated that the excluded group had more participants with an unknown mother's education than predicted by the model compared to the included group.

#### Sex differences

Table 1 summarizes the sex differences across demographic variables. Males were slightly older, *t*_(702)_ = 3.2, *p* = 0.001, and had a higher aerobic fitness level, *t*_(702)_ = 12.0, *p* < 0.001, than females. Pubertal timing was differentially distributed between males and females, with more females reporting stages 3 and 4 compared to more males in stages 2 and 5, *X*^2^ (4, *N* = 704) = 32.30, *p* < 0.001.

#### Correlation analyses and mean RT and accuracy

Table 2 summarizes the Spearman's rank order correlation analyses for inclusion of covariates in step 1 of the regression analyses (Tables 3–**5**). Table 2 also contains mean RT and accuracy information for all outcome information for reference.

**Table 3:** Summary of Hierarchical Regression Analyses for Flanker Reaction Time and Accuracy.

	**Congruent**		**Incongruent**		**Interference score**	
**Model and variable**	**Δ*R^2^***	**β**	**Δ*R^2^***	**β**	**Δ*R^2^***	**β**
**REACTION TIME**						
Step 1	0.053^**^		0.047^**^		0.008	
Age		−0.182^**^		−0.178^**^		−0.088*
Sex		−0.016		0.008		0.035
Pubertal timing		−0.071		−0.063		
Step 2	0.008^*^		0.014^**^		0.009^*^	
Aerobic Fitness		−0.103^*^		−0.135^**^		−0.105^*^
**ACCURACY**						
Step 1	0.037^**^		0.050^**^		0.030^**^	
Age		0.194^**^		0.217^**^		−0.156^**^
Sex		−0.019		−0.084^*^		−0.096
Step 2	0.009^*^		0.010^*^		0.005	
Aerobic fitness		0.106^*^		0.115^*^		−0.082

*Only significant predictors*.

* *p < 0.05*.

** *p < 0.01*.

### Aerobic fitness and flanker task performance

Table 3 summarizes the regression results for RT and accuracy. The regression analyses (unstandardized betas are presented) showed that greater aerobic fitness was associated with better congruent and incongruent performance (*ps* < 0.05), independent of the significant demographic factors entered into step 1. Aerobic fitness was negatively associated with RT interference score, indicating that higher aerobic fitness was associated with less interference (*p* = 0.029). No such associations were found for interference accuracy (*p* = 0.084).

**Table 5** summarizes the flanker diffusion regression results for RT and accuracy. The regression analysis demonstrated that greater aerobic fitness was associated with greater drift rate (i.e., faster acquisition of information) in congruent and incongruent trials (ps < 0.05). No associations were found for boundary separation or nondecision time with aerobic fitness (all *p*s ≥ 0.05).

### Aerobic fitness and switch task performance

Table 4 summarizes the switch regression results for RT and accuracy. The regression analysis showed that in the homogeneous blocks, higher aerobic fitness was associated with shorter RTs (*p* < 0.001), but no such association was found with accuracy (*p* = 0.151). Within the heterogeneous blocks higher aerobic fitness was associated with better accuracy performance in both non-switch and switch trials (*ps* < 0.05). However, longer RT was associated with higher aerobic fitness during switch trials (*p* = 0.018); whereas accuracy was associated with higher fitness, but the associations did not reach significance (*p* = 0.149), indicating the possibility of a speed-accuracy tradeoff. Analyses of the local switch cost found that aerobic fitness was positively associated with RT cost (*p* = 0.005) but not accuracy (*p* = 0.508). Additionally, greater aerobic fitness was associated with greater global switch cost for RT (*p* = 0.002), whereas greater aerobic fitness was associated with reduced global switch cost for accuracy (*p* < 0.001), further supporting the notion of a speed-accuracy tradeoff strategy.

**Table 4:** Summary of Hierarchical Regression Analyses for Switch Reaction Time and Accuracy.

	**Homogeneous**		**Non-switch**		**Switch**		**Global switch cost**		**Local switch cost**	
**Model and variable**	**Δ*R^2^***	**β**	**Δ*R^2^***	**β**	**Δ*R^2^***	**β**	**Δ*R^2^***	**β**	**Δ*R^2^***	**β**
**REACTION TIME**										
Step 1	0.034^**^		0.022^**^		0.016^**^		0.016^*^		0.009^*^	
Age		−0.140^**^		−0.011		0.021		0.045		0.037
Sex		0.054		−0.014		−0.053		−0.053		−0.090
Pubertal timing		−0.064		−0.142^**^		−0.125^*^		−0.132^*^		
Step 2	0.029^**^		0.004		0.011^*^		0.019^*^		0.015^**^	
Aerobic fitness		−0.194^**^		0.071		0.117^*^		0.156^**^		0.139^**^
**ACCURACY**										
Step 1	0.047^**^		0.035^**^		0.047^**^		0.018^**^		0.005	
Age		0.191^**^		0.189^**^		0.217^**^		−0.117^**^		−0.059
Sex		−0.124^**^		−0.032		−0.011		−0.052		−0.032
Step 2	0.004		0.043^**^		0.047^**^		0.042^**^		0.001	
Aerobic fitness		0.069		0.234^**^		0.244^**^		−0.232^**^		−0.033

*Only significant predictor variables included in the models*.

* *p < 0.05*.

** *p < 0.01*.

Table 5 summarizes the switch diffusion regression results. The regression analysis demonstrated that greater drift rate was associated with higher aerobic fitness in both non-switch and switch trials (*ps* < 0.05), indicating faster quality information processing. Positive associations were found between higher aerobic fitness and boundary separation indicating more conservative response patterns for both non-switch and switch trials for higher fit individuals (*ps* < 0.05), further supporting a speed-accuracy tradeoff strategy associated with higher aerobic fitness. Greater nondecision time (i.e., greater time spent during encoding, memory access, and response execution) was associated with higher aerobic fitness in the switch trials (*p* = 0.006), but not in the non-switch trials (*p* = 0.699).

**Table 5:** Summary of Hierarchical Regression Analyses for Flanker and Switch diffusion.

	**Congruent**		**Incongruent**		**Non-switch**		**Switch**	
**Model and variable**	**Δ*R^2^***	**β**	**Δ*R^2^***	**β**	**Δ*R^2^***	**β**	**Δ*R^2^***	**β**
**DRIFT**								
Step 1	0.89^**^		0.093^**^		0.049^**^		0.093^**^	
Age		0.299^**^		0.304^**^		0.239^**^		0.304^**^
Sex		−0.017		−0.077		−0.005		0.014
Pubertal timing						−0.032		
Step 2	0.015^**^		0.015^**^		0.029^**^		0.036^**^	
Fitness		0.139^**^		0.139^**^		0.193^**^		0.215^**^
**BOUNDARY SEPARATION**								
Step 1	0.004		0.024^**^		0.008		0.011	
Age		0.066		−0.106^*^		0.062		0.058
Sex		−0.009		−0.015		−0.070		−0.092^*^
Pubertal Timing				−0.068				
Step 2	0.000		0.003		0.026^**^		0.013^**^	
Fitness		−0.005		−0.067		0.184^**^		0.129^**^
**NONDECISION TIME**								
Step 1	0.029^**^		0.004		0.019^*^		0.012	
Age		0.020		0.058		−0.062		0.060
Sex		−0.032		0.013		0.101^*^		0.007
Pubertal timing		−0.175^**^				−0.084		−0.131^*^
Step 2	0.000		0.000		0.000		0.014^**^	
Fitness		−0.013		−0.012		−0.023		0.136^**^

*Only significant predictors*.

* *p < 0.05*,

** *p < 0.01*.

## Discussion

The aim of this study was to investigate how aerobic fitness is associated with cognitive control in the understudied population of adolescents. The current findings indicate that aerobic fitness was associated with overall higher inhibitory control performance (Table 3). These findings support our *a priori* hypothesis that aerobic fitness would be associated with better performance on tasks of inhibitory control, and converges with previous findings in preadolescents (Buck et al., 2008; Hillman et al., 2009a; Chaddock et al., 2010, 2011; Pontifex et al., 2011; Voss et al., 2011; Scudder et al., 2014; Westfall et al., 2017) and adults (Dustman et al., 1990; Voss et al., 2010; Prakash et al., 2011; Dupuy et al., 2015; Gauthier et al., 2015; Guiney et al., 2015). Furthermore, diffusion modeling analyses helped to elucidate which specific aspects (i.e., drift rate, boundary separation, and/or nondecision time) of the behavioral outcomes were associated with aerobic fitness, affording better characterization of the relationship. Consonant with our hypotheses the results showed that drift rate was particularly sensitive to differences in aerobic fitness for inhibitory control, demonstrating that higher aerobic fitness was associated with improved drift rate, or improved rate of quality information uptake (Table 5). The switching task showed a more nuanced pattern of results (Table 4). When no cognitive flexibility manipulations were present (homogeneous blocks), RT was negatively associated with aerobic fitness. This was similar to non-switch trials such that higher aerobic fitness was associated with improved accuracy. However, in the more difficult switch trials that required more cognitive flexibility, a speed-accuracy tradeoff emerged such that higher aerobic fitness was associated with a conservation of RT in order to improve accuracy (Table 4). The diffusion model also confirms this speed-accuracy tradeoff (Table 5). Boundary separation was particularly sensitive to higher aerobic fitness in cognitive flexibility performance, demonstrating that higher aerobic fitness was associated with more conservative response patterns.

### Inhibitory control

Inhibitory control findings indicated that higher fitness was associated with both higher accuracy and shorter RT in both the congruent and incongruent conditions of the flanker task (Table 3). Accuracy has previously been associated with health behaviors (e.g., physical activity, aerobic fitness, obesity) in children (Chaddock-Heyman et al., 2014; Khan et al., 2014), which is unsurprising as they are more likely to sacrifice accuracy in favor of RT (Davidson et al., 2006). In contrast, previous literature on adolescents is less conclusive with Huang et al. (2015) only finding RT results, Stroth et al. (2009) reporting no relationship, and Hogan et al. (2013) finding fitness-related differences for response accuracy. However, it is important to consider differences in the methods used to measure inhibitory control in these studies. Only Huang et al. used a similar version of the flanker task, whereas the other two studies used a task that combined multiple different inhibitory control requirements (i.e., a modified task that combined elements of Go/NoGo and flanker tasks).

Interestingly, investigations of interference scores indicated a negative association between fitness and RT cost in adolescence, similar to the findings reported by Huang et al. (2015). Such a pattern of results suggests that higher aerobic fitness was associated with improved inhibitory control, as it was associated with less RT interference. The diffusion modeling of the flanker task allowed for further investigation of latent variables that contribute to inhibitory control performance (Table 4). The findings revealed that drift rate in both congruent and incongruent conditions were positively associated with greater aerobic fitness, such that greater aerobic fitness was associated with faster and better quality information accrual. Previous diffusion modeling research has indicated that preadolescents typically have worse drift rate than adults (Ratcliff et al., 2012), and as such, the present results suggest that higher-fit adolescents may express more adult-like patterns of inhibitory control processing. Further, because RT is the summation and decision and nondecision time and because there were no associations between nondecision time and aerobic fitness it seems that faster decision time contributed to the shorter RT associated with higher fitness.

### Cognitive flexibility

Results from the switch task found that higher fitness levels were associated with shorter RT in the homogeneous task (Table 4). Additionally, higher fitness was associated with better accuracy performance in the heterogeneous task for both switch and non-switch trials. Interestingly, higher fitness was associated with longer switch RT performance, indicating that in the more difficult switch trials, requiring higher amounts of cognitive flexibility, higher-fit adolescents increased their RT to improve accuracy. Additionally, higher fitness was associated with larger switch costs in RT for both global and local switch measures. However, at the same time higher fitness was associated with lower global switch cost accuracy, indicating that higher-fit adolescents are utilizing a speed-accuracy tradeoff strategy similar to their adult counterparts (Ratcliff et al., 2012). This pattern was not reflected for local switch cost accuracy, possibly reflecting a move toward response conservativeness associated with higher aerobic fitness.

The diffusion model in the cognitive flexibility task supports a speed-accuracy tradeoff (Table 5). Specifically, higher fitness was associated with a larger boundary separation in both trial types, which is thought to indicate a more conservative response strategy. As indicated previously, this response strategy has been found more common of adults compared to preadolescents (Ratcliff et al., 2012). Additionally, higher fitness was associated with higher drift in both the switch and non-switch trials indicating a more efficient rate of quality data processing. An association between higher aerobic fitness and nondecision time in the switch trials indicates that higher aerobic fitness was associated with more time spent in encoding, memory access, and/or response execution. However, since there was no association between aerobic fitness and RT in the switch trials, greater nondecision time did not affect RT performance. As such, drift rate seems particularly sensitive to differences in aerobic fitness in measures of inhibitory control and cognitive flexibility. Furthermore, boundary separation is sensitive to differences in aerobic fitness, supporting a speed-accuracy tradeoff strategy in measures of cognitive flexibility. As no previous studies have focused on aerobic fitness and diffusion modeling in adolescents, future research is needed to verify our results.

Unexpectedly, results from our study demonstrated differences in which behavioral outcomes were associated with aerobic fitness. Flanker inhibitory control findings indicated that higher fitness was associated with both higher accuracy and shorter RT. Whereas, the cognitive flexibility switch task was related to higher accuracy with greater aerobic fitness, while a speed-accuracy tradeoff was also observed within the switch trials. As mentioned above, when cognitive control demands are increased, children tend to sacrifice accuracy to maintain RT whereas adults demonstrate a slowing of RT in order to maintain accuracy (Davidson et al., 2006). Results from the present study indicate that, in adolescence, aerobic fitness is associated with different behavioral outcome measures between inhibitory control and cognitive flexibility. In other words, in the inhibitory control task, higher aerobic fitness was associated with a smaller sacrifice in RT to maintain accuracy (sacrificing RT to maintain accuracy is a response strategy associated with adults; Davidson et al., 2006). In contrast, higher aerobic fitness resulted in a smaller sacrifice in accuracy to maintain RT in the cognitive flexibility task non-switch trials (sacrificing accuracy to maintain RT is a response strategy associated with preadolescents; Davidson et al., 2006). However, both RT and accuracy were associated with aerobic fitness in the switch trials in the cognitive flexibility task, demonstrating a speed-accuracy tradeoff strategy in the switch trials, which require higher amounts of cognitive flexibility. Such results are not surprising given the developmental differences in the brain from preadolescents to adolescents, and again from adolescents to young adults (Lenroot and Giedd, 2006; Luna, 2009).

## Study strength and limitations

To the best of our knowledge, this study is the first to investigate inhibitory control and cognitive flexibility in adolescents using diffusion modeling techniques. The study includes a relatively large sample, consisting of two cognitive control tasks and various demographic surveys, making it possible to investigate more than one aspect of cognitive control and adjust for possible known confounders. However, there are also several limitations. First, as this study is observational and cross-sectional, no inference of causality can be made. Future studies would benefit from a longitudinal approach to investigate how aerobic fitness levels are associated with changes in cognitive control over time. Randomized controlled trials aiming at increasing aerobic fitness levels in adolescents would further help to elucidate these associations and explore any causal relationships. Both the fitness test and the tests of cognitive control applied in this study are associated with measurement errors, which, if non-differential, can lead to lower β values, which could have influenced our results. However, these β values are similar to those in previously published papers (Scudder et al., 2014; Westfall et al., 2017). Finally, studying a more diverse sample, with a larger range of aerobic fitness levels, may help to further tease apart the associations of aerobic fitness and cognitive control in adolescents.

## Conclusions

In conclusion, the current study demonstrated a beneficial relationship between aerobic fitness and cognitive control in adolescents. Specifically, these findings indicated that higher fitness levels were associated with better inhibitory control and cognitive flexibility. To the best of our knowledge, no previous studies have investigated the associations between aerobic fitness and cognitive flexibility in adolescents. Additionally, this study emphasizes the usefulness of signal-processing methods such as diffusion modeling to combine RT and accuracy to better understand underlying cognitive processing. A particularly interesting finding was that better performance in both RT and accuracy were associated with aerobic fitness in the inhibitory control task, whereas response accuracy was predominately associated with aerobic fitness in the cognitive flexibility task. This result might indicate development toward a more adult-like inhibitory control strategy and a less adult-like cognitive flexibility strategy (Davidson et al., 2006). Further, the diffusion methods indicated that improved quality information uptake (drift rate) was associated with higher aerobic fitness for both inhibitory control and cognitive flexibility. In addition to improved information uptake (drift rate), response conservativeness (boundary separation) was associated with higher aerobic fitness for cognitive flexibility. These findings add additional support to the growing body of literature indicating the importance of aerobic fitness to cognitive health throughout the lifespan.

## Ethics statement

This study was carried out in accordance with the recommendations of Regional Committees on Health Research Ethics for Southern Denmark with written informed consent from all parents/legal guardians. All parents/legal guardians gave written informed consent in accordance with the Declaration of Helsinki. The protocol was approved by the Regional Committees on Health Research Ethics for Southern Denmark (Project number: S-20080047 and S-20140105).

## Author contributions

DW, AK, CH, and AB were primarily responsible for the analysis and interpretation of data presented in the manuscript. AG, JT, NW, and AB were primarily responsible for design and acquisition of the work. DW was primarily responsible for drafting the manuscript and AG, JT, NW, AK, CH, and AB critically revised the draft of the manuscript. All authors provided approval for publication of this content and agree to be accountable for all aspects of the work.

### Conflict of interest statement

The authors declare that the research was conducted in the absence of any commercial or financial relationships that could be construed as a potential conflict of interest.
